# Mapping the prevalence and socioeconomic predictors of low birth weight among Bangladeshi newborns: evidence from the 2019 Multiple Indicator Cluster Survey

**DOI:** 10.1093/inthealth/ihab048

**Published:** 2021-08-04

**Authors:** Md Sabbir Ahmed

**Affiliations:** Department of Community Health and Hygiene, Faculty of Nutrition and Food Science, Patuakhali Science and Technology University, Dumki, Patuakhali-8602, Bangladesh

**Keywords:** Bangladesh, global health, low birth weight, newborn

## Abstract

**Background:**

Low birth weight (LBW) remains a global concern for childhood morbidity and mortality. This study examined the socioeconomic factors associated with LBW among Bangladeshi newborns and drew a district-level prevalence map.

**Methods:**

Data were extracted from the 2019 Multiple Indicator Cluster Survey for Bangladesh. Multivariate logistic regression was used to identify the predictors of LBW.

**Results:**

The prevalence of LBW among Bangladeshi newborns was found to be 14.5%. Overall, the Eastern and South-Eastern regions had a higher burden of LBW. Mothers’ educational status, mode of delivery, wealth index quintile of the household and area were identified as independent predictors of newborns’ LBW. Mothers who completed primary and secondary education grades had a 1.6- and 1.3-fold higher possibility of having an LBW baby compared with those who completed higher secondary or higher educational grades (adjusted OR=1.62 and 1.32, 95% CI 1.21 to 2.18 and 1.06 to 1.65, respectively). Children belonging to the poorest households and residing in urban areas had a 1.4-fold higher likelihood of being LBW (p<0.05).

**Conclusions:**

This study indicates that LBW is still highly prevalent in Bangladesh. Immediate public health action is required in the highly prevalent regions identified in this study.

## Introduction

Low birth weight (LBW) (i.e. birth weight <2500 g) is a global public health concern and a major determinant of infant mortality and morbidity.^1,2^ LBW is further categorized into very low birth weight (<1500 g) and extremely low birth weight (<1000 g).^3^ LBW may occur due to intrauterine growth restriction and/or preterm birth.^4^ Globally, about 15–20% of newborns (i.e. about 20 million) are born with LBW.^5^ The prevalence and incidence of LBW varies significantly across high- and low-income countries, with the majority (95.6%) of newborns with LBW found in low- and middle-income countries.^2^ Evidence shows that in regions of South Asia the rate of LBW is double that of the global percentage.^6^ The higher burden of LBW is an obstacle to achieving Sustainable Development Goal (SDG) targets related to neonatal and child mortality reduction.^7^

A study based on the 2012–2013 Bangladesh Multiple Indicator Cluster Survey (MICS) reported that about 20% of infants were born with LBW.^2^ According to the latest National Low Birth Weight Survey of Bangladesh in 2015, about 22.6% of total infants were born with LBW.^8^ A significant regional variation in the prevalence of LBW was also reported in the earlier study, where the lowest rate was observed in the Rajshahi division (11%) and the highest rate in the Rangpur division (28%).^2^ Previous studies demonstrated severe health consequences of LBW in the early and later life of a child. LBW may cause mortality, diarrhea and respiratory illness in childhood and chronic non-communicable diseases in adulthood.^9–11^ Besides, LBW is also significantly associated with cognitive development and decision-making.^12^ Several factors have been identified as determinants of LBW, such as gestational age, early marriage, less utilization of antenatal care (ANC) services, active or passive smoking and drug abuse, maternal nutritional deficiencies, maternal education and household wealth status.^2^

As a lower-middle-income country, Bangladesh has made substantial improvements in reducing maternal and child mortality; however, the burden of LBW is still very high and is a leading cause of child mortality. This higher LBW rate may hold up the country's improvement in reducing child mortality followed by achieving SDG targets. Previous statistics also indicate an inconsistency in the trend of LBW in Bangladesh.^2,8^ Given the high prevalence of LBW, previous national data on LBW appeared 4 y ago. A national prevalence map of LBW for Bangladesh is also lacking. Therefore, it is essential to fill this research gap and report the latest statistics that could assist in initiating immediate public health action. Further identification of the associated factors and pocket areas are crucial for the policymakers and public health activists to understand where progression has been made and where to allocate additional resources. This study, therefore, aimed to assess the prevalence and associated socioeconomic factors of LBW among newborns. This study also aimed to draw a district-level map based on the prevalence of LBW to identify the pocket areas.

## Methods

### Data source

The present study analyzed the national presentative data of the latest MICS in 2019.^13^ This cross-sectional survey was part of a six-round global MICS conducted by the Bangladesh Bureau of Statistics (BBS) with support from UNICEF. This survey aimed to capture various health indicators and household characteristics (HHs) from district-level samples. Data were collected from January to June 2019. Datasets are freely available at the MICS website (https://mics.unicef.org/surveys). However, to access/download datasets, a formal request of access is required. The author received authorization from the MICS to download and use datasets.

### Sampling design and sample size

Survey data were collected from all 64 administrative districts of Bangladesh. MICS employed a two-stage stratified random sampling procedure to collect data at the household level. The urban-rural areas within each district were considered as the main sampling strata. A specific number of census enumeration areas (EAs) were systematically selected with probability proportional to size within each stratum. After listing HHs within selected EAs, a systematic sample of 20 HHs was drawn from each primary sampling unit (PSU). The total number of PSUs and final sample sizes for this survey were 3220 and 64 400, respectively. For this study, the women's data file was used. Complete data of 64 378 women were available in the dataset, among whom 9183 women had ≥1 live birth in the last 2 y. Data on birth weight (in grams) were available for 4762 children and were considered for analysis in this study.

### Data collection

Data of each individual were collected from the respective mothers from selected households through one-to-one interviews. For this study, women who gave live birth in the last 2 y of the survey date and children weighed at birth were included. Data on birth weight (measured) were collected from birth cards.

### Fieldwork quality control

The supervisor of each data collection team was responsible for the daily monitoring of field data collection. During fieldwork, each team was visited multiple times by survey management team members and UNICEF team members. Field check tables were produced weekly for analysis and action with field teams.

### LBW

Newborns’ birth weight was the outcome variable for this study, which was categorized as a binary variable following the WHO definition^14^: (1) LBW (birth weight <2500 g) and (2) normal weight (birth weight ≥2500 g).

### Study variables

Demographic and socioeconomic status (SES) of the children were considered as independent variables for this study. Independent variables were selected based on a previous study that used the 2012–2013 Bangladesh MICS dataset.^2^ Study variables included: (1) gender of the child (male or female); (2) age of the mother (15–49 y); (3) educational status of the mother (the highest educational level or grade achieved); (4) ANC received (utilization of perinatal/ANC services at least once); (5) ANC assisted by (qualification of the service provider); (6) watch television every day (yes or no); (7) place of delivery (where the mother gave birth to her child); (8) mode of delivery (cesarean or vaginal); (9) wealth index quintile (computed by principal component analysis based on household assets and materials used to build house); (10) area (type of place of residence); and (11) division (of the eight administrative divisions of Bangladesh).

### Data analysis

Pearson's χ^2^ test was performed to compare the outcome variable (i.e. LBW) across different independent variables. Multivariate binary logistic regression analysis was performed to identify the predictors of LBW and to estimate the adjusted OR (AOR) with 95% CI. Variables showing p<0.25 in bivariate analysis were entered into the adjusted regression model.^15^ The tests for the main effects were considered significant at a 5% level of significance. Data were analyzed using SPSS for Windows version 23 (IBM, Armonk, NY, USA) using sampling weight to ensure the results at the national level. Arc GIS v. 10.5 software was used for the graphical distribution of the prevalence of LBW across the 64 districts.

## Results

The data of 4762 (weighed) children, for whom birth weight had been measured after birth, were included in this analysis. Demographics and SES of the participants according to the prevalence of LBW are presented in Table 1. The prevalence of LBW among newborns was found to be 14.5%. Prevalence was found to be significantly higher among those newborns whose mothers had preprimary or no formal education (17.9%), had delivered by the natural vaginal method (16.9%), resided in urban areas (17.6%) and the Chattogram division (19.0%).

**Table 1.** Characteristics of children based on the prevalence of low birth weight

Variable	Frequency (%)	Low birth weight (%)	p^a^
Gender of the child			
Male	2563 (53.8)	13.7	0.092
Female	2199 (46.2)	15.5	
Age of mother (y)			
15–19	673 (14.1)	17.1	0.058
20–24	1622 (34.1)	13.9	
25–29	1316 (27.6)	12.8	
30–34	819 (17.2)	16.7	
35–39	272 (5.7)	14.0	
40–49	60 (1.3)	15.0	
Educational status of mother			
Preprimary or none	180 (3.8)	17.9	<0.001
Primary	693 (14.6)	18.9	
Secondary	2591 (54.4)	14.8	
Higher secondary+	1298 (27.3)	11.1	
Received ANC			
Yes	4501 (94.5)	14.4	0.359
No	261 (5.5)	16.5	
ANC assisted by			
Healthcare professional	4260 (89.5)	14.2	0.060
Other	502 (10.5)	17.3	
Watch television every day			
No	1691 (35.5)	15.2	0.333
Yes	3071 (64.5)	14.2	
Place of delivery			
Home	307 (6.4)	17.5	0.122
Health facility	4455 (93.6)	14.3	
Mode of delivery^b^			
Cesarean	3127 (70.3)	13.2	0.001
Vaginal	1321 (29.7)	16.9	
Wealth index quintile			
Poorest	495 (10.4)	17.8	0.131
Second	701 (14.7)	14.3	
Middle	906 (19.0)	12.7	
Fourth	1138 (23.9)	14.1	
Richest	1522 (32.0)	15.0	
Area			
Urban	1323 (27.8)	17.6	<0.001
Rural	3439 (72.2)	13.3	
Division			
Barishal	182 (3.8)	14.3	<0.001
Chattogram	927 (19.5)	19.0	
Dhaka	1361 (28.6)	15.9	
Khulna	649 (13.6)	10.6	
Mymensingh	255 (5.3)	11.8	
Rajshahi	577 (12.1)	11.4	
Rangpur	547 (11.5)	13.3	
Sylhet	264 (5.5)	13.6	

Abbreviation: ANC, antenatal care.

a χ^2^.

b variable has missing cases.

**Figure 1.**
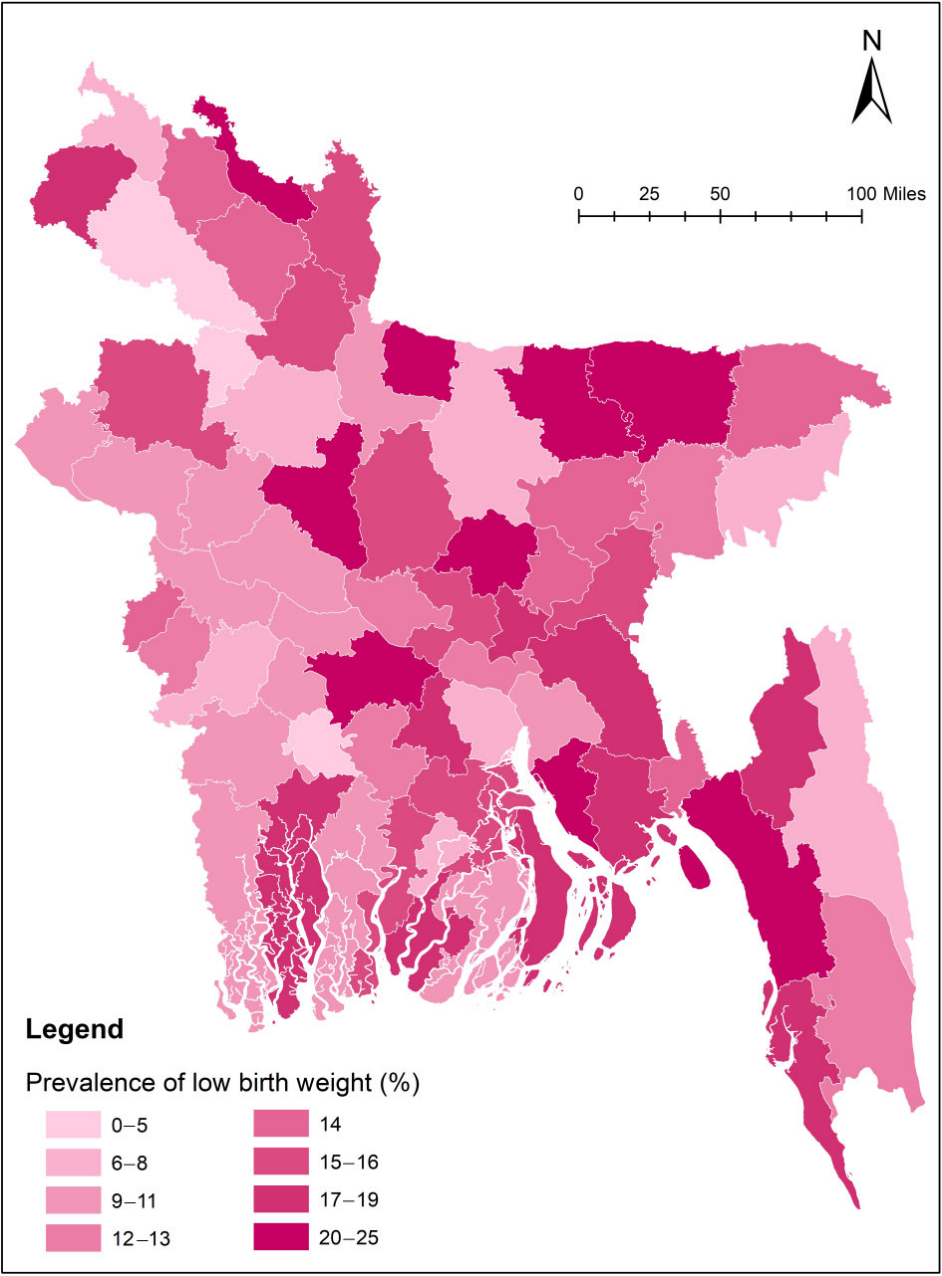
Graphical distribution of the prevalence of low birth weight across the 64 administrative districts in Bangladesh.

Among the 64 administrative districts, Sherpur had the highest burden of LBW (25.0%), which means that one in every four children are born with LBW. This analysis also shows that none of the newborns from the Jaipurhat district had LBW. District-wise prevalence of LBW is presented in Supplementary Table 1. Overall, Eastern and South-Eastern Bangladesh had a higher burden of LBW (Figure 1).

Table 2 shows the adjusted logistic regression analysis of LBW and the SES of the children. After adjusting with potential covariates, educational status of the mother, mode of delivery, wealth index quintile of the household and area were identified as independent predictors of newborns’ LBW. Those mothers who completed primary and secondary education grades had a 1.6- and 1.3-fold higher possibility of having an LBW baby compared with those mothers who completed higher secondary or higher educational grades (AOR=1.62 and 1.32, 95% CI 1.21 to 2.18 and 1.06 to 1.65, respectively). Children delivered by cesarean section had an 18% less probability of being LBW (AOR=0.82, 95% CI 0.68 to 0.99). Mothers who belonged to the poorest households had 1.4 times the possibility of having an LBW baby compared with those who belonged to the richest households (AOR=1.4, 95% CI 1.00 to 2.02). A significant disparity was observed among the urban-rural resident strata. Children from urban areas had 1.4 times the possibility of LBW compared with their rural counterparts (AOR=1.42, 95% CI 1.15 to 1.74).

**Table 2.** Predictors of low birth weight among Bangladeshi newborns

Variable	AOR^†^	p	95% CI
Gender of the child			
Male	0.86	0.086	0.72 to 1.02
Female	Ref.		
Age of mother (y)			
15–19	1.48	0.328	0.671 to 3.29
20–24	1.06	0.876	0.488 to 2.31
25–29	0.97	0.955	0.44 to 2.13
30–34	1.33	0.470	0.61 to 2.92
35–39	0.97	0.947	0.41 to 2.25
40–49	Ref.		
Educational status of mother			
Preprimary or none	1.40	0.177	0.85 to 2.29
Primary	1.62	0.001*	1.21 to 2.18
Secondary	1.32	0.013*	1.06 to 1.65
Higher secondary+	Ref.		
ANC assisted by			
Healthcare professional	0.87	0.363	0.65 to 1.16
Other	Ref.		
Place of delivery			
Home	0.81	0.389	0.50 to 1.30
Health facility	Ref.		
Mode of delivery			
Cesarean	0.82	0.041*	0.68 to 0.99
Vaginal	Ref.		
Wealth index quintile			
Poorest	1.42	0.046*	1.00 to 2.02
Second	1.06	0.691	0.77 to 1.48
Middle	0.98	0.907	0.74 to 1.30
Fourth	1.03	0.816	0.80 to 1.31
Richest	Ref.		
Area			
Urban	1.42	0.001*	1.15 to 1.74
Rural	Ref.		
Division			
Barishal	1.03	0.907	0.58 to 1.83
Chattogram	1.48	0.055	0.99 to 2.22
Dhaka	1.21	0.338	0.81 to 1.81
Khulna	0.77	0.262	0.48 to 1.21
Mymensingh	0.80	0.446	0.46 to 1.40
Rajshahi	0.82	0.422	0.52 to 1.31
Rangpur	0.81	0.389	0.50 to 1.30
Sylhet	Ref.		

Abbreviations: ANC, antenatal care; AOR, adjusted OR; Ref., Reference group.

* Significant p value (p<0.05).

† Adjusted with all the variables in this table.

## Discussion

This study calculated the prevalence and associated factors of LBW from a national representative cross-sectional survey. Current prevalence of LBW was lower compared with the earlier statistics in Bangladesh.^2,8^ This study found that about one in every seven newborns has the possibility of being LBW in Bangladesh. In other words, about 14.5% of newborns are at risk of the consequences of LBW in early and later life.^9–12^ Prevalence of LBW among Bangladeshi newborns was lower than India (16.4%)^16^ and Nepal (15.4%)^17^; however, the prevalence was higher than African countries, for example, Burkina Faso (13.4%), Ghana (10.2%), Malawi (12.1%) and Uganda (10.0%).^18^

A significant regional variation across the country was observed in the prevalence of LBW. This study found that the Chattogram division had the highest (19.0%), while the Khulna division (10.6%) had the lowest rate of LBW, whereas the previous study based on the 2012–2013 MICS reported that the Rajshahi division had the lowest and the Rangpur division had the highest rate of LBW.^2^ Among the 64 administrative districts, 9 were identified as high-risk zones where the prevalence of LBW was >20%, and only 3 had a prevalence <5%. This variation indicates a gap in the existing maternal and child care service system across the country and a low level of awareness of LBW's consequences among the communities in those regions.

Regarding the risk factors, educational status of the mother, mode of delivery, wealth index and type of place of residence (i.e. area) were identified as independent predictors of LBW among Bangladeshi newborns. The likelihood of having an LBW child is negatively associated with the educational status of the mother (i.e. the lower the educational status, the higher the likelihood), which was consistent with earlier studies.^2,16^ Lower educational attainment may limit a mother’s understanding of the consequences of LBW for the newborn and the significance of healthcare-seeking behavior for safe and nourished childbirth.^19^ A previous study also identified women's education as an independent predictor of ANC in Bangladesh.^20^ All these factors could adversely affect fetal growth and increase a woman's risk of delivering an LBW baby.^7^ From the practical viewpoint, it is not feasible to increase formal education to mothers at a later stage of life; however, health education on obstetrical care can certainly be improved. Those children who were delivered by cesarean section had less possibility of being LBW. A previous Ethiopian study reported similar statistics.^21^ The pathology behind this association is not clear. One possible explanation for this could be that because women with higher academic grades who belong to the rich/richest wealth strata mainly have cesarean deliveries in Bangladesh,^22^ they might better understand the consequences of LBW and receive sufficient care during pregnancy. Children from the poorest households had a higher likelihood of being LBW, which is also in line with the earlier study.^2^ Children from urban areas had higher odds of being LBW compared with children from rural areas. It is evident that urban areas had adequate availability and accessibility to healthcare facilities and civic benefits compared with rural areas. However, increased industrialization and environmental pollution in urban areas may adversely affect birth weight (i.e. LBW), which is supported by the previous finding.^23^

This study is not without limitations. The major limitation of this study is the cross-sectional nature of the data. Therefore, a temporal relationship cannot be established. We failed to include all the potential risk factors for LBW, including genetic and environmental factors.^24–27^ Regarding the limitations, the study findings can be generalized at the national level, which is a major strength of this study. In addition to this, the district-level prevalence map from this study could be an essential precursor for future experimental trials and for designing an informed policy-oriented public health program incorporating effective interventions to reduce LBW in Bangladesh.

## Conclusions

About one in every seven newborns are delivered with LBW in Bangladesh. Educational status of the mother, mode of delivery, wealth index quintile of the household and area were identified as independent predictors of newborns’ LBW.

## Supplementary Material

ihab048_Supplemental_File

## Data Availability

Data set used in this study is freely available at https://mics.unicef.org/surveys. However, access to data set requires registration and is granted only for legitimate research purpose.
