# Supplementary material for: Mapping the prevalence and socioeconomic predictors of low birth weight among Bangladeshi newborns: evidence from the 2019 Multiple Indicator Cluster Survey
Source: Int Health. 2021 Aug 4;14(5):485–91. doi: 10.1093/inthealth/ihab048 (PMC9450642; doi:10.1093/inthealth/ihab048)
Supplement: ihab048_Supplemental_File [file ihab048_supplemental_file.docx]

S1. District wise distribution of the prevalence of low birth weight in Bangladesh, MICS-2019

| Sl no. | Division | District | Prevalence of Low birth weight (%) |
| --- | --- | --- | --- |
| 1 | Barishal | Barishal | 15.00 |
| 2 |  | Bhola | 18.80 |
| 3 |  | Barguna | 17.60 |
| 4 |  | Jhalakathi | 8.30 |
| 5 |  | Patuakhali | 10.80 |
| 6 |  | Pirojpur | 15.20 |
| 7 | Chittagong | Bandarban | 11.10 |
| 8 |  | Brahmanbaria | 15.40 |
| 9 |  | Chandpur | 10.70 |
| 10 |  | Chittagong | 24.60 |
| 11 |  | Cumilla | 16.80 |
| 12 |  | Cox’s Bazar | 17.50 |
| 13 |  | Feni | 13.80 |
| 14 |  | Khagrachari | 18.20 |
| 15 |  | Lakshimipur | 22.00 |
| 16 |  | Noakhali | 17.20 |
| 17 |  | Rangamati | 8.30 |
| 18 | Dhaka | Dhaka | 15.60 |
| 19 |  | Faridpur | 23.70 |
| 20 |  | Gazipur | 23.10 |
| 21 |  | Gopalgonj | 12.80 |
| 22 |  | Kishoreganj | 13.70 |
| 23 |  | Madaripur | 16.70 |
| 24 |  | Manikgonj | 11.50 |
| 25 |  | Munshigonj | 12.50 |
| 26 |  | Narayangonj | 17.50 |
| 27 |  | Narshindi | 14.30 |
| 28 |  | Rajbari | 10.00 |
| 29 |  | Shariatpur | 6.50 |
| 30 |  | Tangail | 15.90 |
| 31 | Mymensingh | Mymensingh | 7.40 |
| 32 |  | Jamalpur | 8.80 |
| 33 |  | Sherpur | 25.00 |
| 34 |  | Netrokona | 22.20 |
| 35 | Khulna | Bagerhat | 9.30 |
| 36 |  | Chouadanga | 12.20 |
| 37 |  | Jessore | 10.20 |
| 38 |  | Jhenaidah | 6.70 |
| 39 |  | Khulna | 18.20 |
| 40 |  | Kustia | 9.90 |
| 41 |  | Magura | 10.00 |
| 42 |  | Meherpur | 13.90 |
| 43 |  | Narail | 3.70 |
| 44 |  | Shatkhira | 8.80 |
| 45 | Rajshahi | Bogra | 7.10 |
| 46 |  | Jaipurhat | 0.00 |
| 47 |  | Naogaon | 15.80 |
| 48 |  | Natore | 9.10 |
| 49 |  | ChapaiNawabganj | 10.80 |
| 50 |  | Pabna | 10.80 |
| 51 |  | Rajshahi | 10.70 |
| 52 |  | Sirajganj | 23.40 |
| 53 | Rangpur | Dinajpur | 4.60 |
| 54 |  | Gaibandha | 15.80 |
| 55 |  | Kurigram | 16.10 |
| 56 |  | Lalmonirhat | 23.10 |
| 57 |  | Nilphamari | 14.00 |
| 58 |  | Panchagrah | 6.30 |
| 59 |  | Rangpur | 13.10 |
| 60 |  | Thakurgaon | 17.30 |
| 61 | Sylhet | Hobiganj | 11.30 |
| 62 |  | Moulovibazar | 5.80 |
| 63 |  | Sunamgonj | 21.70 |
| 64 |  | Sylhet | 13.30 |

MICS = Multiple Indicator Cluster Survey
